# Treating ER-positive breast cancer: a review of the current FDA-approved SERMs and SERDs and their mechanisms of action

**DOI:** 10.3389/or.2025.1564642

**Published:** 2025-04-10

**Authors:** Nayoung Kim, Kiven Erique Lukong

**Affiliations:** Biochemistry, Microbiology and Immunology, College of Medicine, University of Saskatchewan, Saskatoon, SK, Canada

**Keywords:** breast cancer, estrogen receptor, SERMs, SERDS, tamoxifen, toremifene, raloxifene, fulvestrant

## Abstract

Breast cancer is one of the most significant causes of mortality among women and the second most prevalent cancer worldwide. Estrogen receptor (ER)-positive breast cancers are the most common molecular subtype of breast cancer, comprising about 70% of breast carcinoma diagnoses worldwide. Endocrine therapy is the foremost strategy for the treatment of ER-positive breast cancer. In the United States, the Food and Drug Administration (FDA) has approved endocrine therapies for ER-positive breast cancers that include selective estrogen receptor modulators (SERMs), selective estrogen receptor downregulators/degraders (SERDs) and aromatase inhibitors (AIs). The approved SERMS, tamoxifen, toremifene and raloxifene, are the gold-standard treatments. The only FDA-approved SERD available for treating ER and hormone-positive breast cancers is fulvestrant, and various generations of AIs, including exemestane, letrozole, and anastrozole, have also received FDA approval. Herein, we review the major FDA-approved SERMs and SERDs for treating ER-positive breast cancer, focusing on their mechanisms of action. We also explore molecular events that contribute to the resistance of these drugs to endocrine therapies and combinational strategies with drugs such as cyclin-dependant kinases 4/6 (CDK4/6) inhibitors in clinical trials to combat endocrine drug resistance.

## 1 Introduction

Breast cancer is currently the second most prevalent cancer worldwide, second only to lung cancer, as reported in Globocan 2022. There were approximately 2.3 million new cases of breast cancer diagnosed in 2022 alone, and over 660,000 deaths were attributed to these diagnoses (1). In the same year, Europe saw over half a million new cases of breast cancer, while 198,553 cases were reported in Africa. Furthermore, North America reported 306,307 newly diagnosed cases, making breast cancer the most prevalent cancer in both the United States of America and Canada (1) (Table 1).

**TABLE 1:** Globocan 2022 – estimated breast cancer incidence and mortality. The table below lists the estimated number of new breast cancer cases and mortality for select continents and countries in 2022 (1). Adapted from (1).

Continent/Country	Incidence	Mortality
Worldwide	2,296,840	666,103
Asia	985,817	315,309
Europe	557,532	144,439
North America	306,307	49,744
Latin America and Caribbean	220,124	59,876
Africa	198,553	91,252
Oceania	28,507	5,483
China	357,161	74,986
United States of America	274,375	42,900
United Kingdom	58,756	12,122
Nigeria	32,278	16,332
Canada	31,823	6,827
Mexico	31,043	8,195
Australia	21,931	3,393

Breast cancer encompasses a group of diseases originating from the breast and displays both biological and molecular heterogeneity (2, 3). Most breast tumours usually develop from the hyperproliferation of ductal epithelial cells before developing into *in situ* and invasive carcinomas, eventually resulting in metastatic disease (3–5). Histologically, breast cancer is divided into three broad categories: *in situ* carcinomas, invasive carcinomas, and metastatic breast carcinomas (2, 6, 7). *In situ* breast carcinoma is further subclassified into ductal *in situ* carcinoma and lobular *in situ* carcinoma (2, 7, 8). Additionally, the World Health Organization (WHO) Classification of Tumors of the Breast, fourth edition, acknowledges the most common histological subtypes of invasive breast carcinoma include invasive tubular, lobular, cribriform, metaplastic, mucinous, apocrine, papillary, and micropapillary carcinomas (9). Tumour grading for breast carcinomas is conducted using methods such as the Nottingham modification of the Scarff-Bloom-Richardson grading system, which grades the tumour on a scale from I-III based on the degree of variation from healthy breast epithelium for gland formation (tubularity) and nuclear size and shape (pleomorphism) (9, 10).

Breast carcinomas can be assessed for tumour stages based on the Tumour-Node-Metastasis (TNM) system, which is comprised of nine stages (0, IA, IB, IIA, IIB, IIIA, IIIB, IIIC, and IV) based on the different combinations for tumour status (T), regional lymph nodes status (N), and metastasis status (M) (9, 11). The American Joint Committee on Cancer (AJCC) Cancer Staging Manual, eighth edition, assigns the T0 category for tumour status when there is no evidence of primary tumour(s) and the Tis category for ductal carcinoma *in situ*. The T1 category is defined by tumours 2 cm in diameter or less; the T2 category of tumours are >2 cm but ≤5 cm in diameter; and the T3 category is defined as tumours greater than 5 cm in diameter. The T4 category is assigned when breast carcinoma cells invade neighbouring and distant tissues and organs (9, 11). The AJCC Cancer Staging Manual, eighth edition, separates regional lymph node status into pathologic N (pN) and clinical N (cN) categories (9, 11). The pN0 category is defined by the absence of regional lymph node metastasis, while the pN1, pN2, and pN3 categories display malignancy in 1-3 lymph nodes, 4-9 lymph nodes, and greater than 10 lymph nodes, respectively. Clinically, the cN classification comprises the cN, cN(f), and cN (sn) categories, which are assigned for confirmed regional lymph node metastasis by clinical findings, core biopsy or fine-needle aspiration, and sentinel node biopsy, respectively (9, 11). Furthermore, metastasis status is classified into two main categories: M0 and M1. M0 indicates the absence of distant metastasis based on radiographic or clinical evaluations. In contrast, M1 signifies the presence of distant metastasis, which includes metastases detected in distant organs or non-regional lymph nodes with a size greater than 0.2 mm (9, 11).

In addition to histological subtypes, breast cancer is classified into five molecular subtypes: luminal A, luminal B, HER2-positive, basal-like or triple-negative (TNBC), and normal-like breast cancers (12, 13). Both luminal A and luminal B subtypes exhibit the expression of estrogen receptors (ER) and are, therefore, considered to be ER-positive breast cancers. Luminal A breast cancers comprise approximately 30%–40% of all invasive breast cancers and are low in grade. Luminal A subtypes are also progesterone receptor (PR) positive and negative for HER2 receptor expression (12, 13). Luminal B breast cancers account for roughly 20%–30% of invasive breast carcinomas and are typically higher in grade. Luminal B subtypes can present as either PR positive or negative, and HER2-positive or HER2-negative with a higher proliferation (Ki-67) score than Luminal A (12–15). HER2-positive breast cancers constitute 10%–15% of invasive breast cancers and are characterized by the overexpression of the HER2 receptor and can be further subtyped based on the positive or negative expression of ER and/or PR (2, 12, 16). TNBC or basal-like subtypes, hallmarked as ER-negative, PR-negative and HER2-negative, represent approximately 15%–20% of invasive breast carcinomas and tend to be aggressive and high-grade. TNBC is further subtyped based on molecular characteristics, with basal-like being the most common subtype (8, 12, 17, 18). The normal-like subcategory of breast cancer makes up less than 10% of invasive breast cancers. The normal-like subtype is similar to luminal A and is characterized as ER and PR-positive, HER2-negative, and displays a low expression of the Ki-67 proliferation marker (2, 19, 20).

Currently, there are various therapeutic strategies for breast cancer, and treatment options predominantly depend on the subtype. Endocrine therapy is the first line of treatment for ER-positive and hormone-positive breast cancers, which account for approximately 70% of total breast cancer diagnoses. Endocrine therapy options comprise selective estrogen receptor modulators (SERMs), selective estrogen receptor degraders (SERDs), aromatase inhibitors (AIs) or combination therapy of two or more drugs (14, 21, 22). Given the similarity of normal-like breast cancer to luminal A subtypes, it is reasonable to infer that SERMs and SERDs provide therapeutic benefits in this setting. However, further research is needed to determine whether there are distinct responses to these agents within the normal-like subtype compared to other ER-positive breast cancers. Treatment for HER2-positive breast carcinoma is typically by targeting the HER2 receptor using anti-HER2 therapies (23–25). However, *de novo* or acquired resistance to anti-HER2 and hormone therapies is common, and therefore, combination therapy of anti-HER2 or hormone treatments with other agents like CDK4/6 inhibitors, PI3K/AKT/mTOR inhibitors, and immune checkpoint inhibitors are being extensively studied in clinical trials (24, 25). TNBC presents a great challenge for breast cancer treatment due to its heterogeneity, poor prognosis, and limited therapeutic options (26). Currently, the standard of care for treating TNBC is chemotherapy, although current research into PARP (Poly [ADP-ribose] polymerase) inhibitors and immunotherapy provides new and targeted treatments for TNBC (26–28). Metastatic breast cancer presents significant challenges due to its aggressive nature and poor prognosis. Unlike early-stage breast cancer, metastatic disease often lacks specific targeted treatment options and is, therefore, conventionally managed with systemic chemotherapy (29, 30). However, not all chemotherapy agents exhibit the same efficacy, and polychemotherapy—the use of multiple agents—generally yields better treatment responses and longer progression-free survival compared to single-agent chemotherapy. Unfortunately, this approach is frequently associated with increased toxicity and a higher risk of adverse effects, underscoring the need for a balanced treatment strategy that maximizes efficacy while minimizing patient burden (29–31).

The regulation of the development, production, marketing, and sales of pharmaceuticals and medical devices in the United States of America is the responsibility of the Food and Drug Administration (FDA). Established in 1906 with the passage of the Pure Food and Drug Act, the FDA is a federal agency responsible for ensuring the quality, safety, and efficacy of drugs, medical devices, food, cosmetics, and other consumer products in the United States. Through rigorous scientific evaluation and regulatory oversight, the FDA plays a critical role in protecting public health and advancing medical innovation (32, 33). The first drug approved by the FDA for treating breast cancer was the cytotoxic agent methotrexate in 1953. Since then, over 30 drugs have been approved for the treatment of both *in situ* and malignant breast carcinomas (34). Although ER-positive breast cancers are more common, less aggressive, and present a better prognosis than HER2-positive and triple-negative breast cancers, treatment for ER-positive breast carcinoma warrants further research as there are no definitive treatment strategies for therapeutic resistance (22, 35). Drug resistance develops in 30%–50% of ER-positive breast cancer patients treated with FDA-approved endocrine therapies (22). As previously mentioned, these endocrine therapies include SERMs and SERDs which function to target and modify estrogen receptor activity and to degrade and/or reduce the expression of the estrogen receptor (21, 22, 36). This review, therefore, examines the current FDA-approved treatments for ER-positive breast cancers and their limitations, with an emphasis on the mechanisms of action of SERMs and SERDs.

## 2 Estrogen receptor signaling

The ER-positive luminal cancers represent the most prevalent subtype of breast cancer, with nearly 70% of breast tumours overexpressing ER, with or without the progesterone receptor (37). Estrogen is the driving force behind mammary gland development and promotes the growth and survival of normal epithelial cells of the breast as well as mammary tumorigenesis. 17β-Estradiol (E2) is the predominant endogenous estrogen and ER ligand in humans (38). Aromatase (encoded by the *Cyp19*/*CYP19* gene) is the rate-limiting enzyme responsible for the unidirectional conversion of androgens to E2 by aromatization in gonadal and extra-gonadal tissues, and it is essential throughout the lifespan in males and females (39, 40).

**FIGURE 1:**
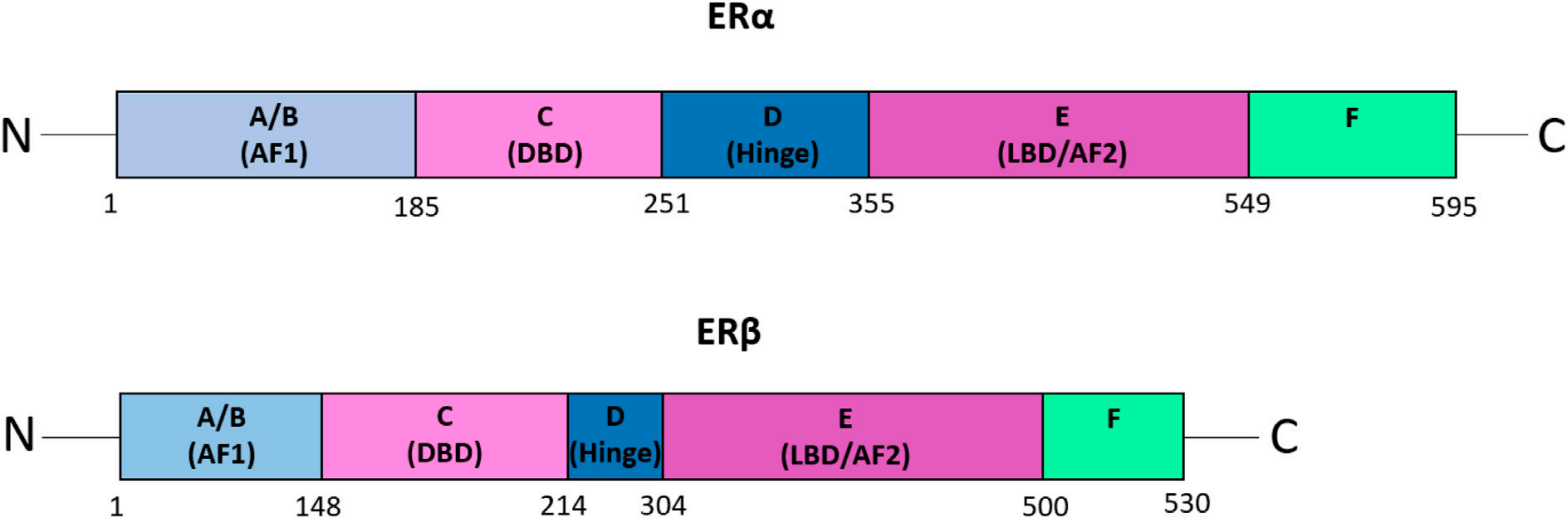
The functional domains of ERα and ERβ. A schematic representation of the domain structure of the estrogen receptors, ERα and ERβ. The five functional domains: A/B, C, D, E, and F, are shown. Domain A/B, also known as the activation factor 1 (AF1) domain, is involved in ligand-independent transcription and interactions with domain E. The C domain is the DNA‐binding domain (DBD), whereas domain D is the flexible hinge region that harbours a nuclear localization signal and the domain for binding heat shock proteins. Domain E, also called the ligand‐binding domain (LBD) or activation factor 2 (AF2) domain, is involved in ligand-dependent transcriptional activation. Furthermore, the LBD possesses a binding site for ligand‐dependent co‐regulator interaction as well as an interface for homo-hetero-dimerization. Domain F is the C-terminal domain, which regulates the transcriptional activation mediated by domains A/B and E (43). Figure adapted from (43).

ERs are members of the nuclear hormone receptor family that include ERα and ERβ, which are encoded by the ESR1 and ESR2 genes and are composed of 595 amino acids and 530 amino acids, respectively (41) (Figure 1). ERα and ERβ share five functional domains: A/B, C, D, E and F (42). The A/B domain, also called the activation factor 1 (AF1) domain, is located in the amino-terminal and is involved in ligand-independent transcription and interactions with domain E. Domain C is the DNA‐binding domain (DBD), while the D domain is a flexible hinge region that harbours a nuclear localization signal and heat shock proteins-binding domain. Domain E is the ligand‐binding domain (LBD), and the activation factor 2 (AF2) domain is also involved in ligand-dependent transcriptional activation. The LBD also harbours an interface for homo-hetero-dimerization and a binding site for ligand‐dependent co‐regulator interaction (43). The C-terminal domain (domain F) regulates the transcriptional activation mediated by domains A/B and E (43).

**FIGURE 2:**
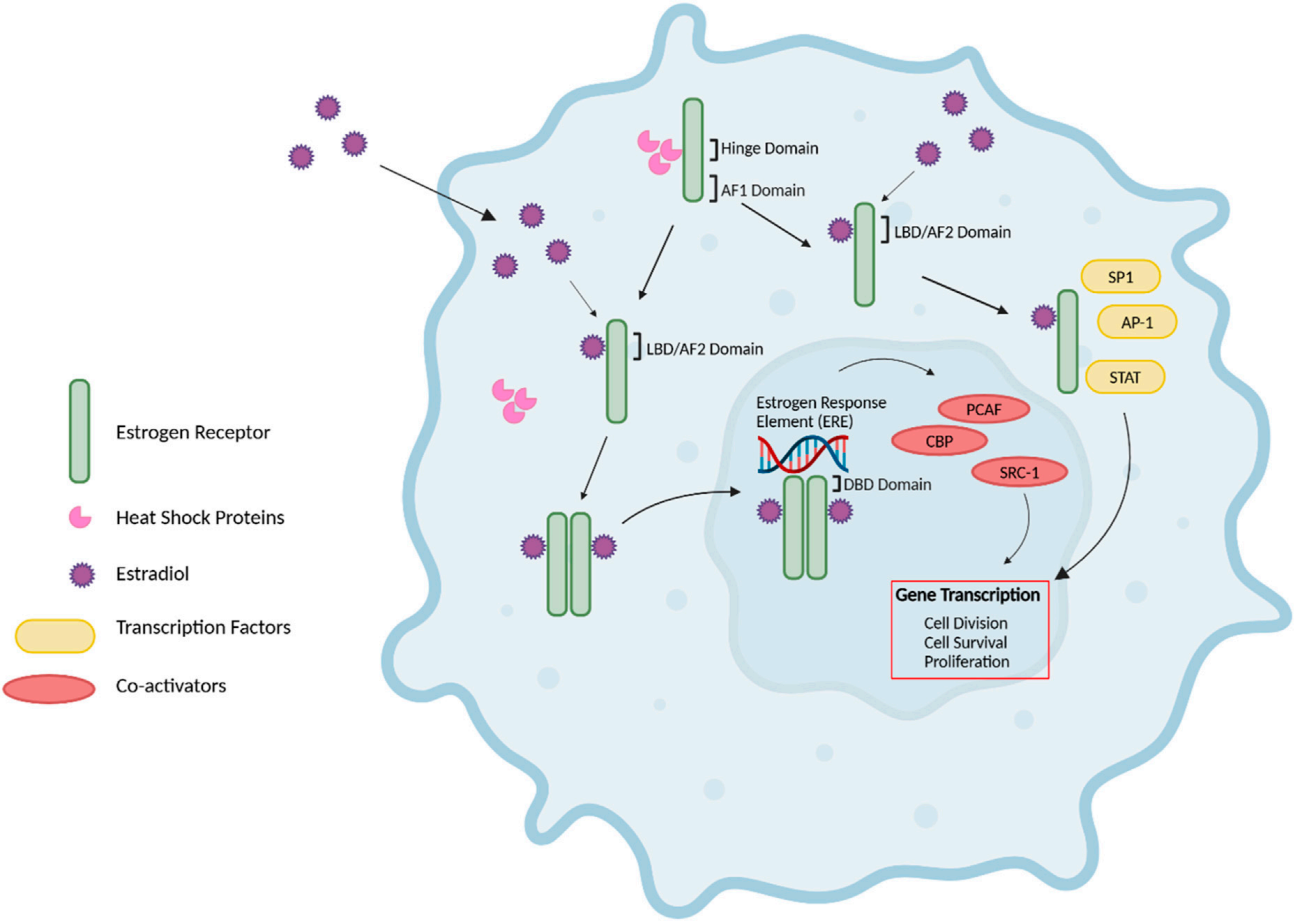
Estrogen Receptor Signaling. The nuclear estrogen response element (ERE) pathway is depicted. 17β-Estradiol (E2), the predominant endogenous estrogen and estrogen receptor (ER) ligand, binds to the ligand binding domain (LBD/AF2) domain of the ER to undergo a conformational change. This conformational change enables the dimerization, activation, and nuclear translocation of the ER to the nucleus, where the ER complex binds to EREs in the promoter region of the target gene via the DNA-binding domain (DBD). Subsequently, this facilitates the recruitment of co-activators such as PCAF and CBP histone acetyltransferases and steroid coactivator-1 (SRC-1), thus promoting various cellular activities, including cell division, survival and proliferation. Further, E2-bound ER can also result in non-classical genomic signaling where liganded ER regulates the expression of genes in an ERE-independent manner through the direct interaction with transcription factors, including specificity protein 1 (SP1), activator protein 1 (AP-1) transcription complex, and the signal transducers and activators of transcription (STAT) family of transcription factors. Without E2, ER monomers remain predominantly in the cytoplasm in an inactive (non-DNA-binding) state, sequestered in multiprotein chaperone complexes organized around heat shock proteins bound to the ER hinge domain. The activation factor 1 (AF1) domain primarily contributes to ligand-independent activation of the ER (43–45, 47, 48). Figure generated using (50).

ERs signal through various pathways, including i) the nuclear estrogen response element (ERE)-dependent pathway, ii) the nuclear ERE-independent pathway, and iii) the extranuclear/estrogen-independent pathway (44) (Figure 2). Without E2, ER monomers remain predominantly in the cytoplasm in an inactive (non-DNA-binding) state, sequestered in multiprotein chaperone complexes organized around heat shock proteins (HSPs), particularly HSP90 (45). It is important to note that while ERα plays a predominant role in driving cell proliferation and survival in ER-positive breast cancer, ERβ has been shown to exert opposing effects by inhibiting cell proliferation and promoting apoptosis (Murphy and Leygue, 2012). Furthermore, studies have demonstrated that ERβ antagonizes ERα-mediated transcriptional activity by competing for ERE binding, recruiting co-repressors, and modulating non-genomic signaling pathways (46).

The nuclear ERE-dependent pathway is the classic ER genomic pathway in which E2-activated ER undergoes a conformational change and dissociates from the multiprotein chaperone complexes, thus releasing HSP90. This conformational change enables the dimerization and activation of the ER. Activated ER translocates to the nucleus, where it binds to EREs in the promoter regions of target genes, facilitating the sequential recruitment of co-activators such as histone acetyltransferases (including CBP/p300 and PCAF) and steroid receptor coactivator-1 (SRC-1), which can robustly enhance the transcription of target genes and promote cellular activities such as cell survival, division, and proliferation (44).

Interactions between E2 and ER can also result in non-classical genomic signaling, whereby the liganded ER regulates the expression of genes in an ERE-independent manner via direct interaction with transcription factors such as the activator protein 1 (AP-1) or specificity protein 1 (SP1) transcription complex, NF-kappaB (NFkB), and the signal transducers and activators of transcription (STAT) family of transcription factors (47, 48).

In the extranuclear/estrogen-independent pathway, ERs may reside in or can be translocated to the cell membrane or cytoplasm, where they can rapidly initiate cellular signaling events by direct interaction with receptor tyrosine kinases such as HER2, EGFR, and insulin-like growth factor-1 receptor (IGF1R) [reviewed in (48)]. Crosstalk between the ER and these receptors can activate the mitogen-activated protein kinase (MAPK) and phosphoinositide 3-kinase (PI3K) pathways, leading to gene expression changes that enhance cell growth [reviewed in (49)]. It is important to note the extent to which extranuclear/estrogen-independent pathway depends on classical ERs is complex. GPER (G protein-coupled estrogen receptor), an alternative membrane estrogen receptor, also binds 17β-estradiol and triggers overlapping signaling cascades, including activation of adenylate cyclase, ERK1/2, and calcium mobilization (50). Furthermore, Prossnitz and Barton describe how GPER functions independently of ERα and ERβ, yet there is evidence of some functional interplay between these receptors (49). Some studies suggest that classical ERs may even modulate GPER activity through direct or indirect interactions (51). The interplay between these pathways warrants further investigation, particularly in the context of endocrine resistance in ER-positive breast cancer.

The intricacy of ER-signaling in mammary gland development adds to the complexity of identifying a single effective therapeutic target for ER-positive breast cancer. However, the ER is targeted therapeutically directly by antiestrogen agents such as the SERM, tamoxifen, the SERD, fulvestrant, and indirectly by the use of aromatase inhibitors that block the production of estrogen (52, 53). Interestingly, fulvestrant and the SERMs tamoxifen and raloxifene have been found to activate the GPER, acting as a GPER agonist (50, 54–60). This information warrants additional research to fully understand the complex role of these drugs in the context and consideration of both the ER and GPER pathways for breast cancer therapy.

## 3 FDA-Approved drugs for ER-positive breast cancer

Current FDA-approved endocrine therapies for ER-positive breast cancers target to modify or decrease ER expression and activity or inhibit estrogen biosynthesis. As previously mentioned, these therapies fall under three main categories: i) selective ER modulators (SERMs), ii) selective ER downregulators/degraders (SERDs) and iii) aromatase inhibitors (AIs). SERMs function to target and modify ER activity, while SERDs degrade and/or decrease the expression of the ER. AIs function by blocking estrogen biosynthesis, thereby reducing the amount of estrogen circulating in the body (21, 22, 36). SERMs, such as tamoxifen, are approved endocrine therapies for premenopausal and postmenopausal women (61, 62). The SERD fulvestrant, warrants further treatment studies in premenopausal women, who are still producing estrogen via ovaries, and is more often used to treat breast cancers in postmenopausal women (34, 63, 64). Aromatase inhibitors on the other hand, are often utilized in postmenopausal women, as this treatment stops estrogen production in the breast of postmenopausal women via inhibition of the aromatase enzyme (62, 65, 66).

### 3.1 Selective estrogen receptor modulator (SERM)

SERMs have been used to treat various diseases and conditions, including breast cancer, osteoporosis and postmenopausal symptoms. Depending on the target tissue, SERMs have the potential to display characteristics of either estrogen agonists or antagonists (67). SERMs are able to interact with both ERα and ERβ to elicit either agonistic effects, such as growth and proliferation when targeting bone tissue, or opposing antagonistic effects in target tissues, such as the breast and mammary epithelia (68, 69). Additionally, selective estrogen receptor modulators have been found to increase the expression and activity of low-density lipoprotein (LDL) receptors, thus reducing LDL cholesterol levels (70, 71). Furthermore, SERMs have been found to inhibit the biosynthesis of cholesterol, resulting in a further contribution to the reduction of LDL cholesterol levels (60, 69, 72–75). Currently, three SERMs have been approved by the FDA for the treatment of ER-positive breast cancers. They include tamoxifen and toremifene, which are triphenylethylene derivatives differing only by the presence of a chlorine atom in the ethyl chain of toremifene, and raloxifene, a benzothiophene derivative (76).

#### 3.1.1 Tamoxifen

##### 3.1.1.1 Approval and use

Tamoxifen is a first-generation breast cancer drug that was approved by the FDA for the treatment of breast cancer in 1977. Originally synthesized as a method of contraception in 1962, tamoxifen is a non-steroidal derivative of triphenylethylene and is one of the most utilized endocrine therapies for ER-positive and hormone-positive breast cancers (77, 78). Various clinical trials have evaluated the efficacy of tamoxifen in the prevention and treatment of breast cancers, including the National Surgical Adjuvant Breast and Bowel Project (NSABP) Protocol B-14 trial in 1981. The Protocol B-14 trial was a randomized clinical study that evaluated the efficacy of tamoxifen in women with ER-positive breast cancer with negative axillary nodes. Participants received either 10 mg of tamoxifen twice daily or a placebo for 4 years following surgery. The results demonstrated that women treated with tamoxifen had a 75% higher likelihood of remaining disease-free compared to those who received the placebo (79). Furthermore, tamoxifen therapy was found to significantly reduce the rates of local and distant treatment failures. Although the trial was effective in prolonging the disease-free survival in those receiving tamoxifen treatment, adverse effects reported more frequently in the tamoxifen group compared to placebo include vaginal discharge, irregular menses, hot flashes, and thromboembolic events (79). Additional side effects include but are not limited to, headaches, dizziness, and depression (80). Further, treatment with tamoxifen has been found to increase the risk of endometrial cancer due to its estrogen agonist effects in the uterus (81–84). Tamoxifen treatment has also been shown to have effects on the ovaries in premenopausal women, such as an increased incidence of benign ovarian cysts, ovarian enlargement, stimulation of ovarian steroidogenesis, and induction of ovulation (85–89). Although tamoxifen displayed biological activity as a full antagonist in the mammary epithelium and is, therefore, an extremely effective treatment for breast cancer, the drug acts as an agonist in other organs, including the endometrium, where it can promote endometrial proliferative disorders, including hyperplasia (90). Tamoxifen is sold under the brand name Soltomax Oral Solution in the United States through pharmaceutical companies such as Fortovia Therapeutics Inc. Likewise, in Canada, AstraZeneca and several other companies sell brand names including Nolvadex-D among others. Tamoxifen is also marketed under various generic names, including tamoxifen citrate in the United States and supplied by companies such as Actavis Pharma, Inc. Additionally, Apo-Tamox tab 10/20 mg is one of the various generic brands of tamoxifen sold in Canada through companies such as Apotex Corporation. Standard tamoxifen dosing is typically 20 mg administered daily, either in tablet or solution form. Furthermore, drug interactions for tamoxifen include but are not limited to, warfarin, aromatase inhibitors such as anastrozole, inducers of CYP3A4, and strong inhibitors of CYP2D6 (80). According to the American Society of Clinical Oncology recommendations, newly diagnosed premenopausal and perimenopausal ER-positive patients take tamoxifen doses daily for 5 years as their first hormonal therapy.

##### 3.1.1.2 Tamoxifen metabolism

**FIGURE 3:**
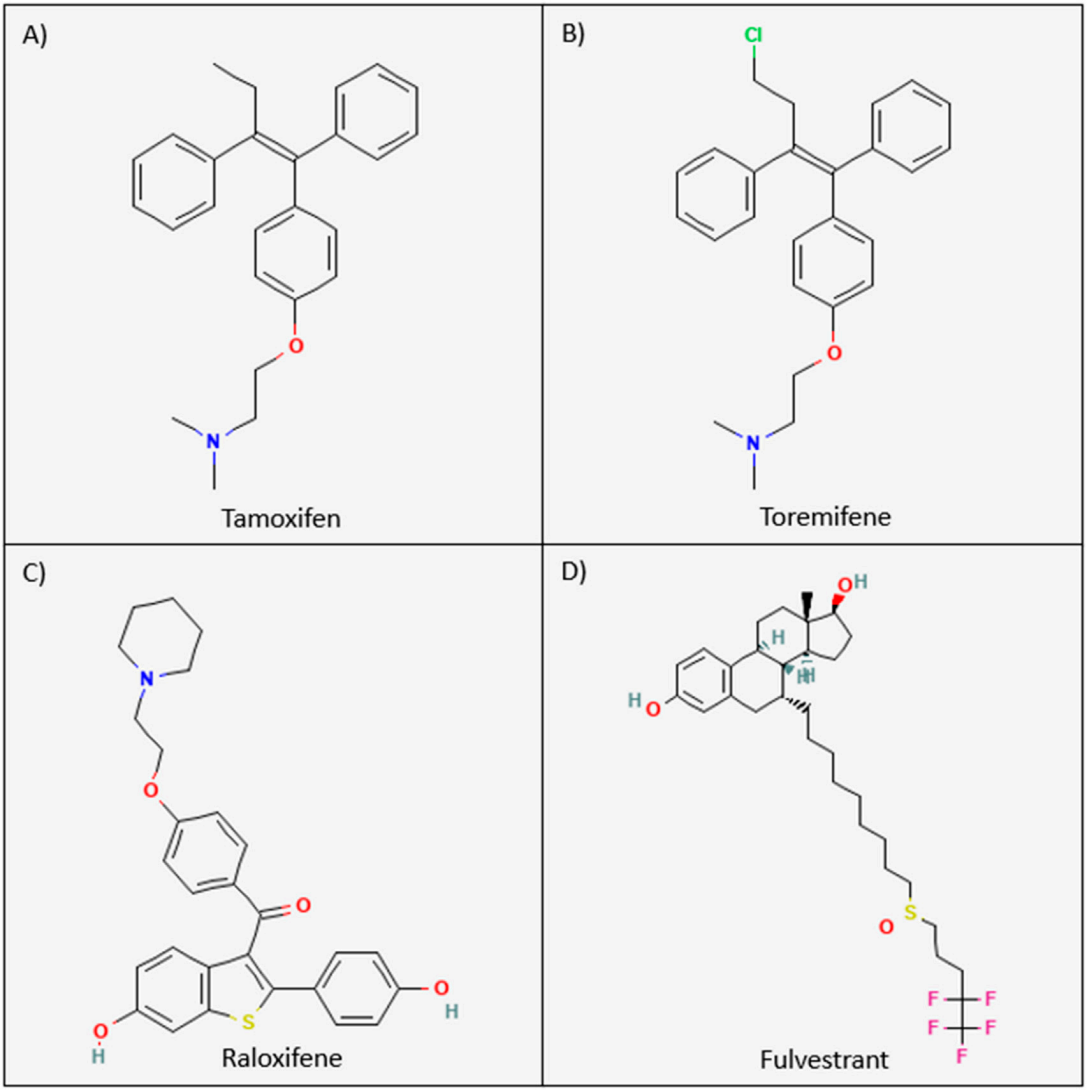
Chemical structures of the current FDA-approved SERMs and SERDs. The chemical structures for tamoxifen, toremifene, raloxifene, and fulvestrant are visualized above. **(A)** Tamoxifen: (Z)-2-[p-(1,2-diphenyl-1-butenyl)phenoxy]N,N-dimethylethylamine. **(B)** Toremifene: 2-[p-[(Z)-4-chloro-1,2¬diphenyl-1-butenyl]phenoxy]-N,N-dimethylethylamine. **(C)** Raloxifene: [6-hydroxy-2-(4-hydroxyphenyl)-1-benzothiophen-3-yl]-[4-(2-piperidin-1-ylethoxy)phenyl]methanone. **(D)** Fulvestrant: 7-alpha-[9-(4,4,5,5,5-penta fluoropentylsulphinyl)nonyl]estra-1,3,5-(10)-triene-3,17¬beta-diol (190).

**FIGURE 4:**
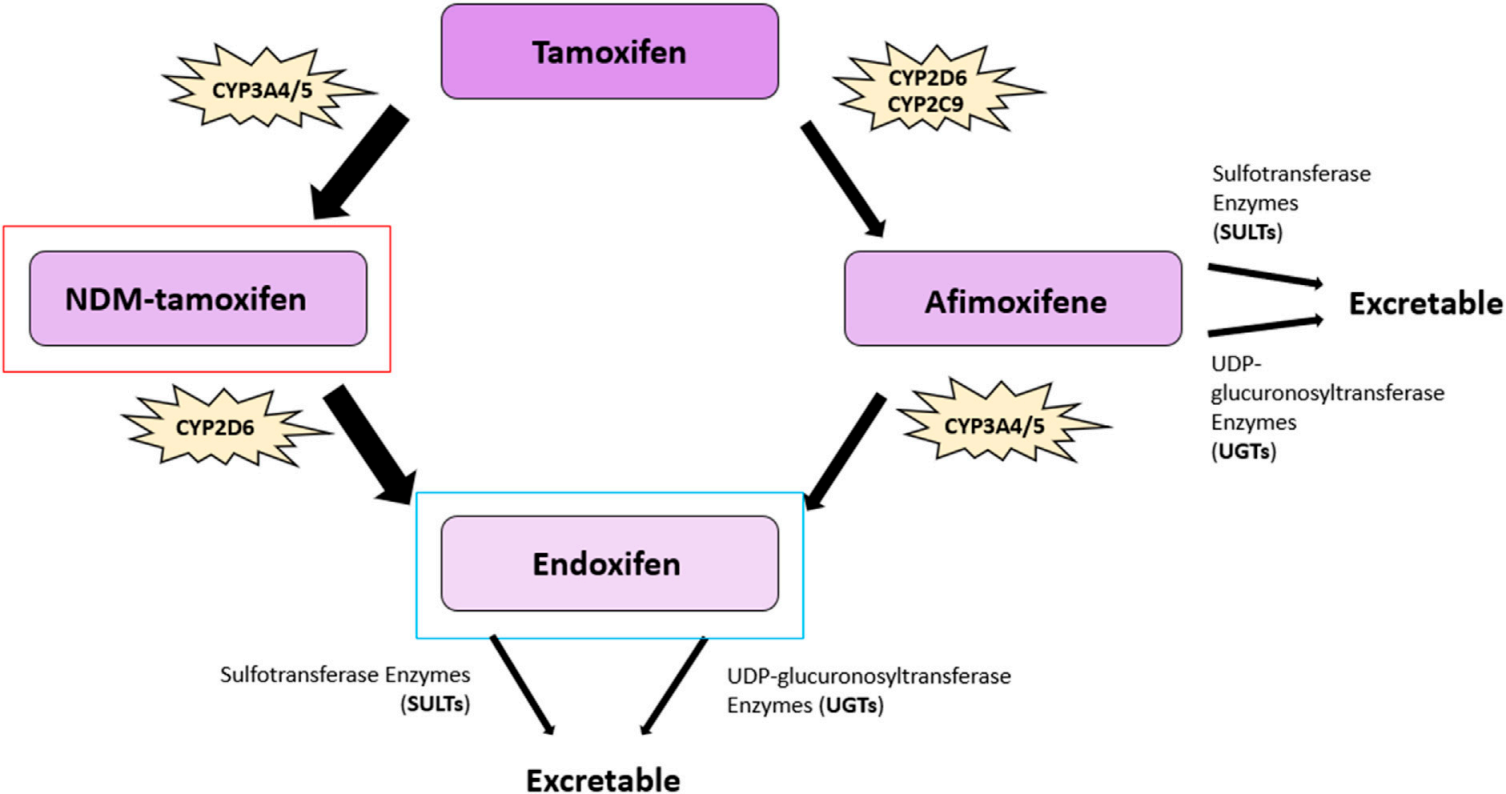
Tamoxifen metabolic pathway. The major metabolites of tamoxifen: 4-hydroxy tamoxifen (afimoxifene), and 4-hydroxy, *N*-desmethyl tamoxifen (endoxifen), are shown, as well as the metabolite N-desmethyltamoxifen (NDM-tamoxifen). The primary active tamoxifen metabolite, endoxifen, is highlighted in blue and NDM-tamoxifen, the major circulating metabolite, is highlighted in red. The conversion of tamoxifen to NDM-tamoxifen constitutes approximately 92% of tamoxifen metabolism, whereas the pathway through 4-OHT represents about 7%. The key cytochrome P450 (CYP) enzymes involved in bioconversion are highlighted alongside the other cytochrome P450 isoenzymes. CYP3A4/5 catalyzes the N-demethylation of tamoxifen to produce NDM-tamoxifen, which is subsequently 4-hydroxylated by CYP2D6 to generate endoxifen. Tamoxifen is converted to afimoxifene by enzymes such as CYP2D6 and CYP2C9 and is further metabolized into endoxifen primarily via CYP3A enzymes. Afimoxifene and endoxifen are converted into excretable forms via sulfotransferase and UDP-glucuronosyltransferase enzymes (92, 93, 99, 100). Figure adapted from (97).

Tamoxifen ((*Z*)-2-[*p*-(1,2-diphenyl-1-butenyl)phenoxy]*N*,*N*-dimethylethylamine) is a non-steroidal SERM exhibiting strong antiestrogen effects in mammary epithelia (91) (Figure 3). Tamoxifen is a prodrug possessing a low affinity for its target, the ER. Tamoxifen is extensively metabolized in the liver by the cytochrome P450 isoforms CYP2D6, CYP3A4, CYP3A5, CYP2C9, and CYP2C19 via two pathways to generate two of its most potent metabolites, afimoxifene (4-hydroxy tamoxifen, 4-OHT) and endoxifen (4-hydroxy, *N*-desmethyl tamoxifen) (92, 93) (Figure 4). The major pathway involves the cytochrome P450 isoenzyme (CYP) 3A4/5 (CYP3A4/5), which catalyzes the N-demethylation of tamoxifen to N-desmethyltamoxifen (NDM-tamoxifen), which is then 4-hydroxylated by the polymorphic CYP2D6 to produce endoxifen. In a separate metabolic pathway, tamoxifen is converted to 4-OHT by many enzymes, including CYP2D6 and CYP2C9 (93). Further, 4-hydroxy tamoxifen tends to lose a methyl group to yield endoxifen in a process catalyzed primarily by CYP3A (92). Although both 4-hydroxy tamoxifen and endoxifen exhibit 30–100-fold greater affinity for the estrogen receptor than the parent drug tamoxifen (94). Endoxifen, which is produced at five to ten times higher concentrations than 4-OHT, is considered the primary metabolite (95). The conversion of tamoxifen to NDM-tamoxifen constitutes about 92% of tamoxifen metabolism, while the pathway through 4-OHT represents only about 7% (96). However, tamoxifen, formulated as tamoxifen citrate, has a half-life of 5–7 days, whereas endoxifen, formulated as z-endoxifen hydrochloride, has a much shorter half-life between 49.0 and 68.1 h (98). Both 4-OHT and endoxifen are converted into excretable forms by the UDP-glucuronosyltransferase enzymes and sulfotransferase enzymes (99, 100). The ATP-binding cassette (ABC) transporters–ABCB1 (P-gp/MDR1), ABCC1 (BRCP), and ABCC2 (MRP2) are known to be elevated in multiple drug resistance and mediate the efflux of metabolites such as 4-OHT and endoxifen (101, 102). ABCB1, for example, is expressed in 28%–63% of breast tumours and has been shown to bind 4-OHT and endoxifen (102, 103).

##### 3.1.1.3 Mechanism of action

**FIGURE 5:**
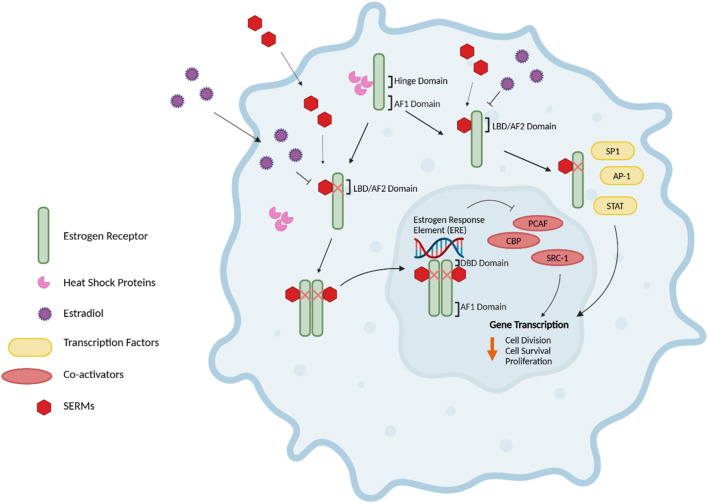
Mechanism of action of SERMs. The selective estrogen receptor modulators (SERMs), tamoxifen, toremifene, and raloxifene bind to the ER like 17β-Estradiol (E2) to induce conformational change. Heat shock proteins (HSP) chaperones, such as HSP90 are released as part of ER activation and nuclear translocation from the ER hinge domain. This results in the dimerization of ER and translocation to the nucleus, preferentially activating the activation factor 1 (AF1) domain while suppressing the activation factor 2 (AF2)/ligand-binding domain (LDB). Secondly, SERM-bound ER will bind to the Estrogen Response Element (ERE) on promotors of target genes, thus resulting in transcription attenuation of E2-responsive genes due to the inactivation of the ligand-dependent AF2 domain and reduced ER co-activator binding. Additionally, SERMs can influence transcription factor pathways involving proteins such as STAT and AP-1 pathways via meditation through altered ER-cofactor interactions (22, 35, 104). Figure generated using (106).

Tamoxifen, a non-steroidal ER antagonist, produces metabolites which competitively bind to the ER and displace estrogen to inhibit its proliferative effects in breast tissue (91) (Figure 5). Like E2, tamoxifen also binds to the ER, albeit with lower affinity, and induces a distinct conformational change that promotes the release of HSP90 and ER dimerization. The ER dimer translocates to the nucleus where it will first promote the activation of the activation factor 1 (AF1) domain and inhibit the activation factor 2 (AF2) domain or ligand-binding, and second, bind to ERE on promotors of target genes (104). Through these processes, the Tamoxifen-ER dimer attenuates the transcription of the E2-responsive genes since the ligand-dependent AF2 domain is inactivated and ER co-activator binding is reduced. Further, tamoxifen-induced ER dimers recruit corepressors such as the HDACs (histone deacetylases) and SMRT (silencing mediator of retinoid acid and thyroid hormone receptor), also known as N-CoR2 (105–107). HDACs, for instance, deacetylate histones that subsequently lead to the inhibition of transcription. The recruitment of the co-repressor proteins is, therefore, pivotal to the antiestrogen effects of tamoxifen in mammary epithelia (105–107).

#### 3.1.2 Toremifene

##### 3.1.2.1 Approval and use

Toremifene is a selective estrogen receptor modulator and a chlorinated derivative of tamoxifen. Toremifene was therefore approved by the FDA in 1997 for the treatment of metastatic breast cancers of ER-positive origin or tumours of unknown ER expression (108, 109). The drug is widely used for the treatment of both early and late stages of metastatic breast cancer (109). Toremifene was approved with the hope that it would display a better safety profile than tamoxifen (108). A study conducted by the International Breast Cancer Study Group found that 76% of patients with ER-positive tumours demonstrated a 5-year disease-free survival after receiving 60 mg of toremifene daily following chemotherapy on day 15 over a 5-year period. Additionally, the 5-year survival of patients with ER-positive breast cancer who received toremifene treatment over a 5-year duration was 90% (110). In the United States, toremifene is sold as tablets under the brand name Fareston and as the generic product, toremifene citrate, through suppliers such as Kyowa Kirin, Inc. and Rising Pharmaceuticals, Inc., respectively. The FDA-recommended dose of toremifene for the treatment of metastatic breast cancer in postmenopausal women is 60 mg once daily. However, doses as high as 120 mg have been approved in countries such as Japan and shown to be effective in treating metastatic breast cancer in patients who have relapsed on aromatase inhibitors (111, 112). Toremifene toxicity of grade 3 or higher was experienced in 7% of patients taking 60 mg of toremifene daily in the study for the International Breast Cancer Study Group (n = 499). Grade 3 toxicity symptoms include vascular events such as deep vein thrombosis and phlebitis pulmonary embolism, myocardial infarction, and congestive heart failure, among others (110). Drug interactions listed by the FDA for toremifene include warfarin, agents that prolong the QT interval, drugs that decrease the excretion of renal calcium, and strong inducers or inhibitors of CYP3A4 (113).

##### 3.1.2.2 Toremifene metabolism

**FIGURE 6:**
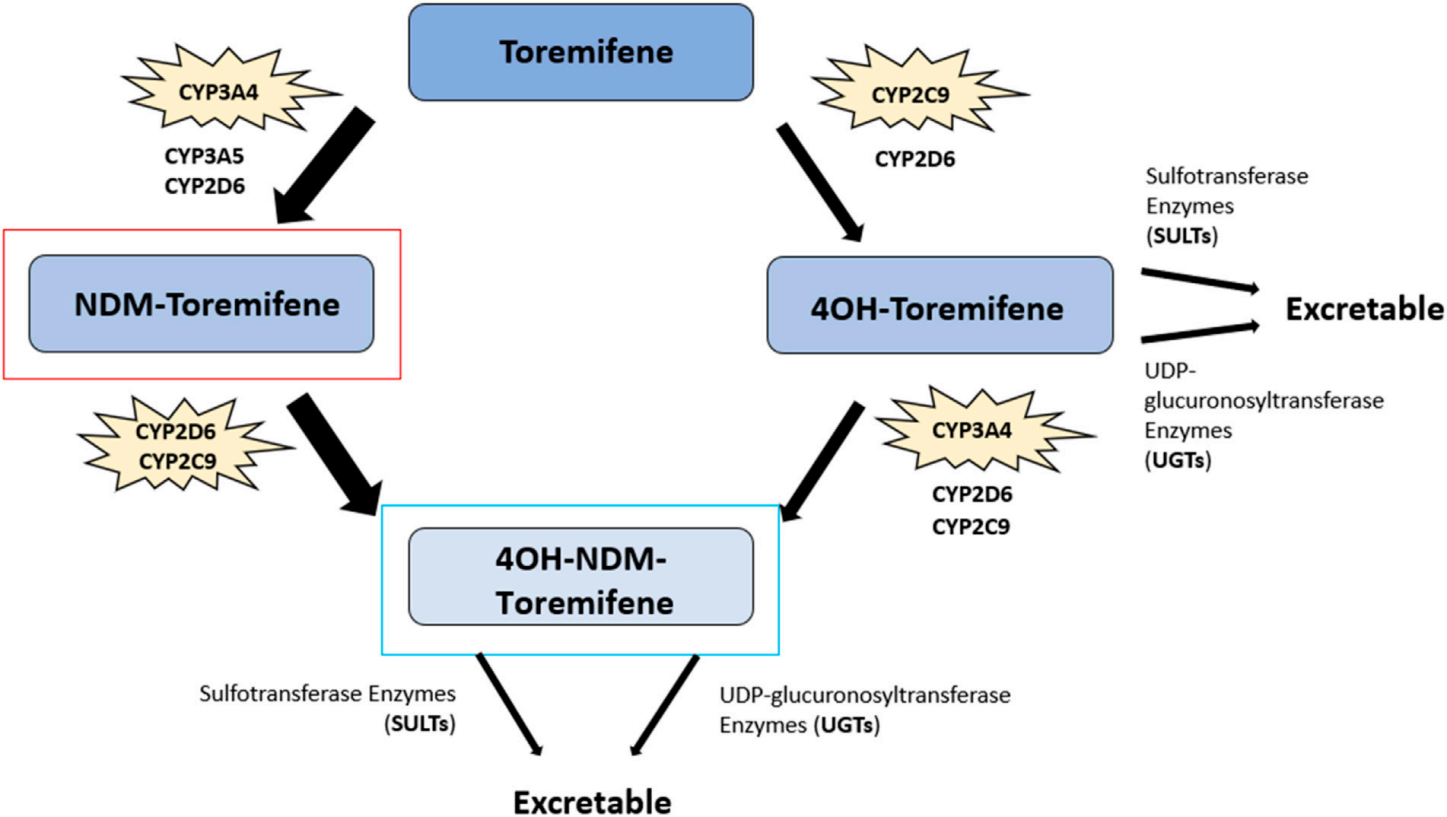
Toremifene metabolic pathway. The major circulating metabolite of toremifene, N-desmethyl (NDM) toremifene, is highlighted in red while the primary active metabolite of tamoxifen, 4OH-NDM-toremifene, is highlighted in blue. 4-hydroxy (4-OH) toremifene, another metabolite, is also shown. The key cytochrome P450 (CYP) enzymes involved in toremifene metabolism are noted alongside other cytochrome P450 isoenzymes. The conversion of toremifene into NDM-toremifene occurs primarily via CYP3A4, with CYP3A5 and CYP2D6 also contributing to a lesser extent. NDM-toremifene is converted into its secondary metabolite, 4OH-NDM-toremifene, through the CYP2D6 and CYP2C9 isozymes. Further, toremifene is converted to 4OH-toremifene mainly via CYP2C9, with CYP2D6 contributing a minor role. The CYP3A4 is the main isozyme in converting 4OH-toremifene into 4OH-NDM-toremifene, with CYP2D6 and CYP2C9 also involved to a lesser degree. 4OH-toremifene and 4OH-NDM-toremifene are converted into excretable forms through sulfotransferase and UDP-glucuronosyltransferase enzymes (100, 117, 118, 122, 123).

Toremifene (2-[p-[(Z)-4-chloro-1,2diphenyl-1-butenyl]phenoxy]-N,N-dimethylethylamine) is structurally similar to tamoxifen, differing in just one chlorine atom substituting a hydrogen atom in the ethyl side chain, but it is equally as effective as tamoxifen for treating breast cancer (114–116) (Figure 3). Toremifene is lipophilic and over 99% bound to plasma proteins such as albumin (117, 118). Like tamoxifen, toremifene is metabolized in the liver to form metabolites, including *N*-desmethyl (NDM) toremifene, 4-hydroxy (4OH) toremifene and 4-hydroxy-*N*-desmethyl (4OH-NDM) toremifene (119) (Figure 6). The major circulating metabolite of toremifene is NDM-toremifene, while levels of 4OH-toremifene, the primary active metabolite, and 4OH-NDM-toremifene are much lower in human plasma (119, 120). In breast cancer cell lines, the 4OH- and 4OH-NDM-toremifene metabolites are approximately 100-fold higher in activity than both toremifene and NDM-toremifene (119). The metabolism of toremifene into its metabolites occurs via two pathways. The first pathway is the conversion of toremifene into NDM-toremifene mainly through the cytochrome P450 isoenzyme CYP3A4, although CYP3A5 and CYP2D6 are also contributors (119, 121). The conversion of NDM-toremifene into its secondary metabolite, 4OH-NDM-toremifene, occurs via the CYP2D6 and CYP2C9 isozymes. The second pathway of toremifene metabolism involves the conversion of toremifene into 4OH-toremifene primarily through the CYP2C9 isoenzyme, with CYP2D6 playing a minor role. The bioconversion of 4OH-toremifene into 4OH-NDM-toremifene occurs mainly through CYP3A4, although CYP2D6 and CYP2C9 are also involved to a lesser degree (119, 121). Due to its similarities to tamoxifen, toremifene metabolites such as 4-OH-toremifene, are also converted into excretable forms through sulfotransferase and UDP-glucuronosyltransferase enzymes (100, 122, 123). The half-life of toremifene and NDM-toremifene is typically 5 days, whereas the half-life for 4OH-toremifene is approximately 6 days (124). Additionally, the concentration of the major metabolite, NDM-toremifene, is greater than toremifene 8 hours after administration (119, 125).

##### 3.1.2.3 Mechanism of action

Toremifene is a non-steroidal SERM exhibiting antiestrogen effects on target tissues such as the mammary epithelia and partial agonist effects in uterine and bone tissues (109, 118). Although equivalent in its estrogen binding and anti-tumour properties as tamoxifen, toremifene may be less genotoxic, as research conducted in rat hepatocytes found lower toremifene DNA adducts compared to tamoxifen (110, 118, 126, 127). Toremifene and its metabolites competitively bind to the ER to displace estrogen, thereby inhibiting the growth and proliferative effects of estrogen on breast tissue (118, 128). The mechanism of action of toremifene is similar to that of tamoxifen (Figure 5). Upon binding to the ER, toremifene and its metabolites initiate ER dimerization in the same fashion as E2. The ER dimer is translocated to the nucleus, leading to the activation of the AF1 domain, the inactivation of the AF2 domain, and the binding of the dimer to the ERE of target gene promoters. The toremifene-ER complex results in a decrease in the binding of ER co-activators in addition to the already inactivated AF2 domain, resulting in the reduced transcription of estrogen-responsive genes (118, 128).

#### 3.1.3 Raloxifene

##### 3.1.3.1 Approval and use

Raloxifene is a second-generation, selective estrogen receptor modulator approved by the FDA for the prevention of postmenopausal osteoporosis in 1997 and for the treatment of osteoporosis in postmenopausal women in 1999. In 2007, the FDA approved raloxifene for the risk reduction of invasive breast carcinoma in postmenopausal women (34, 63). Clinical trials such as the Multiple Outcomes of Raloxifene Evaluation (MORE) were conducted to observe the effectiveness of raloxifene in the risk reduction of breast cancer in postmenopausal women with osteoporosis. The MORE trial, a randomized, multicenter, double-blind trial, was conducted between 1994 and 1998, where 7705 postmenopausal women with osteoporosis across 25 countries were administered raloxifene or placebo and followed up after a median of 40 months. The trial found that the risk of invasive breast carcinoma decreased by 76% during the 3 years that patients received raloxifene treatment. Furthermore, the risk of ER-positive breast cancer was decreased by 90% during the raloxifene treatment (129). In general, raloxifene is well tolerated, although an increase in the rate of hot flashes and leg cramps was reported. Other adverse effects reported in the trial include peripheral edema, endometrial cavity fluid and influenza-like syndromes (129).

Raloxifene is sold in the United States under the brand name Evista by companies such as Physicians Total Care, Inc. and as the generic raloxifene hydrochloride through companies like Actavis Pharma Company. Furthermore, raloxifene is sold in other countries, such as Canada, for the prevention of breast cancer. For breast cancer risk reduction, the standard dosing of raloxifene is 60 mg taken orally, once daily. Interactions listed by the FDA for raloxifene include cholestyramine, warfarin, systemic estrogens, and other highly protein-bound drugs, among others (130).

##### 3.1.3.2 Raloxifene metabolism

**FIGURE 7:**
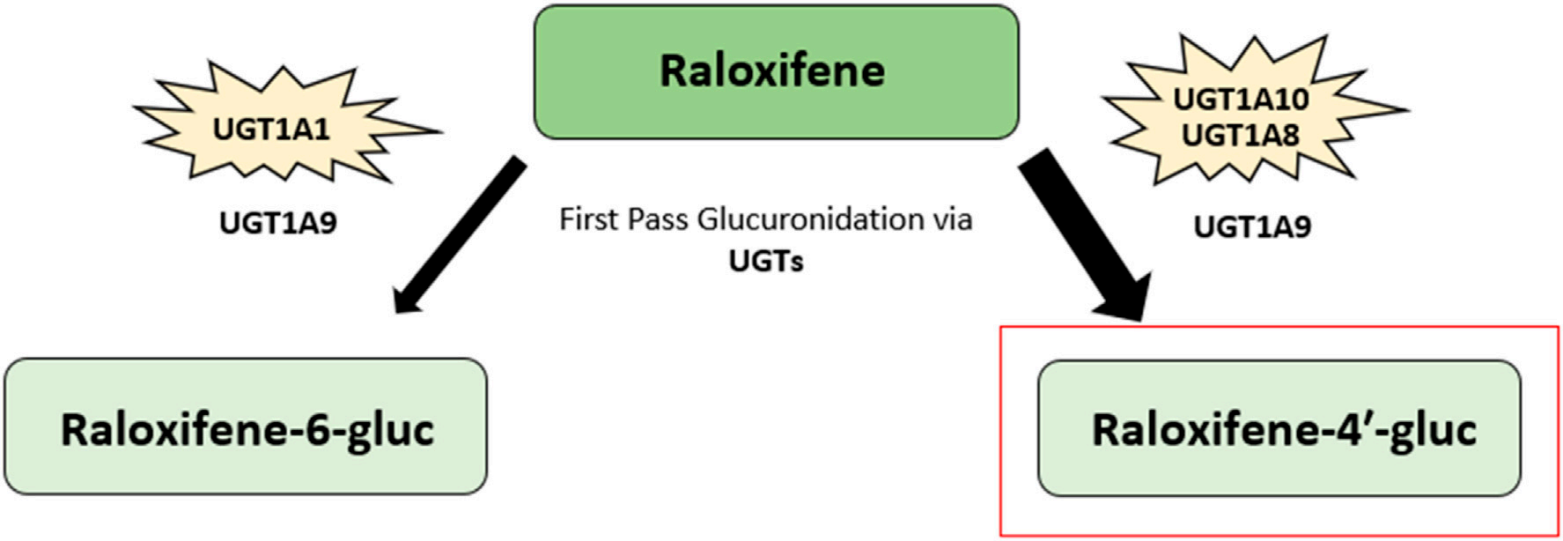
Raloxifene metabolic pathway. The major metabolite of raloxifene, raloxifene-4′-β-glucuronide (raloxifene-4′-gluc), is highlighted in red. Another metabolite, raloxifene-6-β-glucuronide (raloxifene-6-gluc), is also shown. Raloxifene undergoes extensive first-pass glucuronidation into its metabolites upon gastrointestinal tract absorption. The 1A UDP-glucuronosyltransferase (UGT) enzyme family are involved in raloxifene metabolism. Raloxifene is converted to raloxifene-4′-gluc metabolite mainly through intestinal UGT1A10 and UGT1A8 enzymes. The conversion of raloxifene to raloxifene-6-gluc occurs primarily via hepatic UGT1A1. The UGT1A9 enzyme has been found to play a minor role in catalyzing the formation of both raloxifene-4′-gluc and raloxifene-6-gluc (131–135).

Raloxifene ([6-hydroxy-2-(4-hydroxyphenyl)benzo [b]thien-3-yl]-[4-[2-(1-piperidinyl) ethoxy]phenyl]methanone) is a selective estrogen receptor modulator belonging to a class of compounds known as the benzothiophenes (Figure 3). Like tamoxifen and toremifene, raloxifene also exhibits estrogen agonist or antagonist effects on differing target tissues (63). Raloxifene is rapidly absorbed after oral administration, with up to 60% absorbed following administration of a treatment dose. Additionally, the drug does not undergo significant P450-dependent oxidation. Instead, raloxifene undergoes extensive first-pass glucuronidation upon absorption into the gastrointestinal tract, resulting in less than 2% bioavailability (131–133). Upon first pass glucuronidation, raloxifene is converted into the metabolites raloxifene-6-β-glucuronide (raloxifene-6-gluc) and raloxifene-4′-β-glucuronide (raloxifene-4′-gluc) (131, 134) (Figure 7). Raloxifene-4′-gluc is the major metabolite found in human plasma, with an approximate ratio of 8:1 for raloxifene-4′-gluc:raloxifene-6-gluc. Further, only less than 1% of unconjugated raloxifene is found in human plasma (131, 132). Previous research has found the 1A UDP-glucuronosyltransferase (UGT) enzyme family to be involved in the metabolism of raloxifene (135). UGTs are membrane-bound enzymes found in the endoplasmic reticulum and are involved in catalyzing the transfer of glucuronic acid to substrates. The resulting conjugates possess an increase in water solubility (136, 137). The conversion of raloxifene to the raloxifene-4′-gluc metabolite occurs primarily by intestinal UGT1A10 and UGT1A8 enzymes, while the bioconversion of raloxifene to raloxifene-6-gluc occurs mainly through hepatic UGT1A1. Additionally, UGT1A9 was shown to play a minor role in catalyzing the formation of raloxifene-4′-gluc and raloxifene-6-gluc (135). Raloxifene possesses an elimination half-life of approximately 28 h and an apparent oral clearance of 44 L/kg per hour (131, 133).

##### 3.1.3.3 Mechanism of action

Raloxifene is a non-steroidal SERM exhibiting estrogen agonist or antagonist effects depending on the target tissue (63). Estrogenic effects of raloxifene occur in bone and lipid metabolism, whereas antiestrogen effects are exhibited in breast tissue, and neutral effects are observed in the endometrium (63, 67, 138, 139). Raloxifene exhibits a higher affinity for the ER than its glucuronide conjugate metabolites (140). Raloxifene has a similar affinity to the ER as E2 and can initiate ER dimerization and translocation to the nucleus once bound to the ER via its benzothiophene ring (Figure 5). The binding of raloxifene to the ER results in a spatial change in the configuration of the ER, resulting in the activation of the AF1 domain and inactivation of the AF2 domain of the ER. Subsequently, the raloxifene-ER complex binds to the ERE of target gene promoters (141–143). Furthermore, the raloxifene-ER complex results in a decrease in the binding of ER co-activators, thus resulting in the reduced transcription of estrogen responsive genes). Additionally, once raloxifene binds to the ER, the raloxifene-ER complex then recruits coregulators to further enhance the antiestrogen effects of raloxifene (63, 144). However, raloxifene exerts a different effect on other tissues, such as bone. The raloxifene-ER complex, with the aid of various helping, activating, and/or adapting (HP) proteins, is able to bind and activate a specific DNA sequence known as the Raloxifene Responding Element (RRE). The binding of the raloxifene-ER complex to the RRE results in the transcription of genes involved in the synthesis of specific cell proteins responsible for the estrogen growth and proliferative effects of raloxifene on these tissues (141–143).

### 3.2 Selective estrogen receptor downregulators/degraders (SERDs)

As discussed earlier, anti-endocrine therapies such as tamoxifen, a selective ER modulator, and aromatase inhibitors such as anastrozole were FDA-approved in 1977 and 1995, respectively (65, 145). Tamoxifen quickly became the gold standard therapy for advanced and early-stage estrogen-sensitive cancers. However, the potentially serious side effects and other presentations following tamoxifen treatment, such as relapses, exposed the need for other anti-endocrine therapies with fewer or less severe side effects. The advent of selective estrogen receptor downregulators/degraders (SERDs) was, therefore, a welcome addition to antiestrogen therapeutic armamentarium. SERDs are estrogen receptor antagonists employed for treating ER and hormone-positive breast cancers. SERDs exert their anti-tumour effects through the inhibition, downregulation, and degradation of the ER, therefore abrogating the proliferative effects of estrogen in breast cancer cells (21, 146, 147). Fulvestrant is the only SERD approved by the FDA for the treatment of hormone-positive breast carcinomas. The drug is an effective and well-tolerated drug for the treatment of metastatic ER-positive breast cancer.

#### 3.2.1 Fulvestrant

##### 3.2.1.1 Approval and use

Fulvestrant was approved by the FDA in 2002 for the treatment of ER and hormone-positive, metastatic breast cancers in postmenopausal women (34, 148). Fulvestrant is sold in the United States as the brand name Faslodex by companies such as AstraZeneca Pharmaceuticals LP and as the generic fulvestrant through companies like Amneal Pharmaceuticals LLC. Furthermore, fulvestrant is sold in various countries including Canada, France, and Spain. The FDA lists the standard dosing of fulvestrant for the treatment of breast cancer as 500 mg as two intramuscular injections, one for each buttock, administered on days 1, 15, and 29 and subsequent once-monthly doses (149). Additionally, there are no known drug interactions for fulvestrant (150). Clinical trials have been conducted to evaluate the efficacy of fulvestrant for use in hormone-positive breast cancers. Such trials include the FALCON study, a randomized, double-blind, international, phase III clinical trial where 524 patients with hormone-positive, locally advanced or metastatic breast cancer were enrolled between 2012 and 2014 (151). The FALCON trial aimed to evaluate the efficacy of fulvestrant compared to the aromatase inhibitor, anastrozole, in patients who had not received prior endocrine therapy. The trial demonstrated that patients receiving a 500 mg dose of fulvestrant had a longer median progression-free survival than those treated with 1 mg of anastrozole daily, with medians of 16.6 months and 13.8 months, respectively. Therefore, fulvestrant demonstrates comparable, if not superior, efficacy in extending progression-free survival in patients with hormone receptor-positive, locally advanced, or metastatic breast cancer when compared to aromatase inhibitors like anastrozole (151). Although fulvestrant is an effective and generally well-tolerated treatment option, common adverse effects reported in trials such as the FALCON include arthralgia, hot flashes and gastrointestinal disturbances (151, 152).

##### 3.2.1.2 Fulvestrant metabolism

**FIGURE 8:**
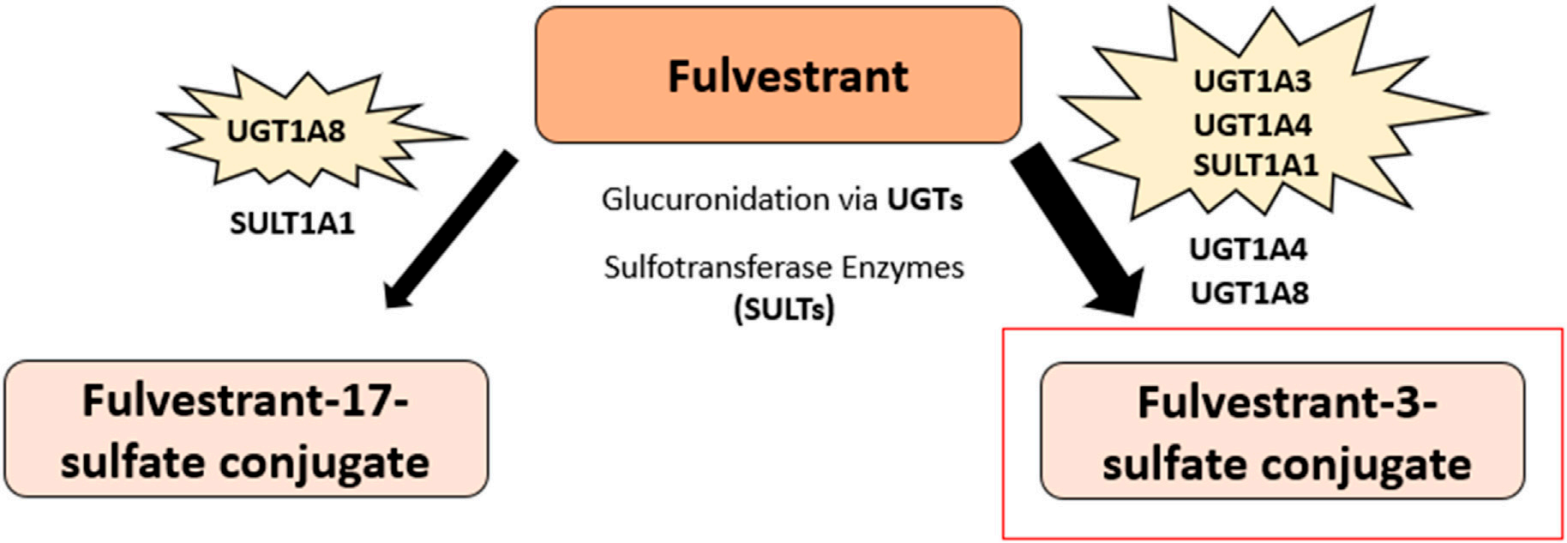
Fulvestrant predominant metabolic pathway. The major metabolite of fulvestrant, fulvestrant-3-sulfate conjugate, is highlighted in red. Fulvestrant glucuronidation at the 3-hydroxyl position and sulfate conjugation produces sulfated-fulvestrant, specifically the metabolite fulvestrant-3-sulfate conjugate. Another metabolite of fulvestrant, fulvestrant-17-sulfate conjugate, is also shown and is produced via fulvestrant glucuronidation at the −17 position and through sulfate conjugation. The 1A UDP-glucuronosyltransferase (UGT) enzyme family are involved in the metabolism of fulvestrant. The majority of fulvestrant is metabolized via UGT1A3 and UGT1A4 enzymes, which catalyze the glucuronidation of fulvestrant at the 3-hydroxyl position. UGT1A1 and UGT1A8 were also found to also play a minor role in the glucuronidation of fulvestrant. Further, fulvestrant can be converted to fulvestrant-17-glucuronide through the UGT1A8 enzyme. Fulvestrant is sulfated by sulfotransferase enzymes, namely SULT1A1, to produce sulfated-fulvestrant (153–155).

Fulvestrant (7-alpha-[9-(4,4,5,5,5-penta fluoropentylsulphinyl)nonyl]estra-1,3,5-(10)-triene-3,17beta-diol) is a 7α-alkylsulphinyl analogue of estradiol that competes with E2 for binding to the ER (148) (Figure 3). Fulvestrant is metabolized in the body via rapid glucuronidation at its −3 and −17 positions and through sulfate conjugation to produce sulfated-fulvestrant (153, 154) (Figure 8). The glucuronidation of fulvestrant is catalyzed by the A1 UDP-glucuronosyltransferase (UGT) family enzymes, namely UGT1A1, UGT1A3, UGT1A4, and UGT1A8 (154). The majority of fulvestrant is metabolized by UGT1A3 and UGT1A4, the main enzymes which catalyze the glucuronidation of fulvestrant at the 3-hydroxyl position, although UGT1A1 and UGT1A8 were also found to also play a minor role. Furthermore, UGT1A8 can convert fulvestrant into fulvestrant-17-glucuronide, although this metabolite only accounts for 5%–10% of total fulvestrant glucuronidation (154). Previous research suggests that fulvestrant may be inactivated in both the liver and intestine due to the high expression levels of the UGT1A3 and UGT1A4 enzymes (154).

Additionally, the cytochrome p450 isoenzymes, CYP1A2, CYP2C9 and CYPA4, may also be involved in the metabolism of fulvestrant, although the sulfate conjugation of fulvestrant is suggested to be the more predominant pathway in comparison (154, 155). Fulvestrant is sulfated via sulfotransferase enzymes, namely SULT1A1, to produce sulfated-fulvestrant, particularly the fulvestrant-3-sulfate conjugate (153, 154). Due to its low bioavailability and pre-systemic metabolism, fulvestrant was designed for administration via intramuscular injection over oral ingestion (156). Fulvestrant has an elimination half-life of approximately 40 days after a 250 mg dose, with an estimated apparent volume of distribution at steady state ranging from 3 to 5 L/kg (155).

##### 3.2.1.3 Mechanism of action

**FIGURE 9:**
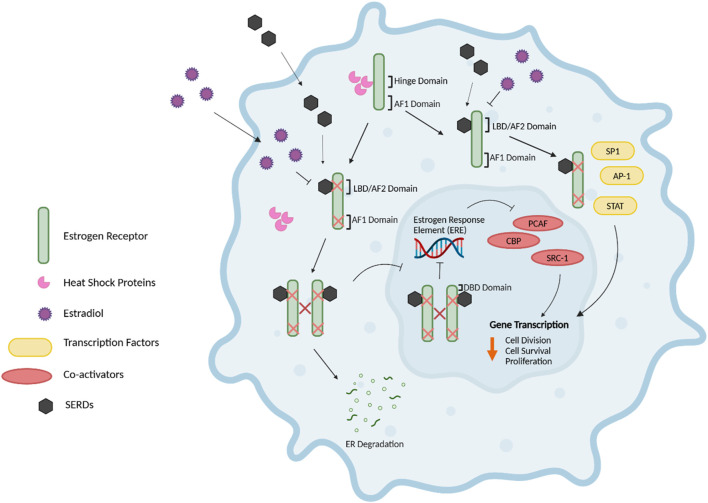
Mechanism of action of SERDs. The selective estrogen receptor downregulator/degrader (SERD), fulvestrant, exerts anti-tumour effects via the inhibition, downregulation, and degradation of the ER, therefore hindering the proliferative effects of estrogen in breast cancer cells. The attenuation of ER dimerization via fulvestrant competitively binding to the ligand binding domain (LBD)/AF2 domain of the ER results in the impairment and inhibition of the fulvestrant-ER complex for nuclear translocation, thus preventing the fulvestrant-ER complex from binding to the estrogen response element (ERE) of target gene promoters. The impaired dimerization of the ER inhibits its activation, as receptor dimerization is critical for ER function and nuclear localization. Additionally, the conformational change of the ER via fulvestrant disrupts both the Activation Factor 1 and 2 (AF2)- and (AF1)-related transcriptional activity. The fulvestrant-ER complex is fragile and unstable, therefore resulting in the accelerated proteasomal degradation of the ER in comparison to the ER bound with estradiol or SERMs such as tamoxifen. Further, SERDs can influence the pathways involving transcription factors such as STAT and AP-1 through the mediation of altered ER-cofactor interactions (21, 158–161, 191). Figure generated using (50).

Unlike SERMs, which exert both estrogen agonist and antagonist effects depending on the target tissue, fulvestrant exhibits purely antiestrogen effects. Fulvestrant has a high affinity for the ER, with an approximate 89% binding affinity to that of estradiol (157). The attenuation of ER dimerization via fulvestrant, results in the subsequent impairment and inhibition of the fulvestrant-ER complex for translocation to the nucleus (158) (Figure 9). Furthermore, the impaired dimerization inhibits the activation of the ER, as receptor dimerization is crucial for ER function and nuclear localization (158, 159). While tamoxifen blocks the E2-mediated activity of the AF2 domain, resulting in ER-antagonistic activity, the fulvestrant-induced conformational change of the ER disrupts both AF2- and AF1-related transcriptional activity (158, 159). Since fulvestrant hinders the ER’s ability for nuclear translocation, the fulvestrant-ER complex thus cannot localize and bind to the ERE of target gene promoters. However, if any fulvestrant-ER complexes are successful in nuclear localization, the complex is still rendered transcriptionally inactive due to the hindered AF1 and AF2 domains (158). Additionally, the fulvestrant-ER complex is unstable and fragile, thus resulting in the accelerated proteasomal degradation of the ER compared to the ER bound with estradiol or tamoxifen (160). The accumulation of the fulvestrant-ER complex in the cytoplasm may also promote its degradation, as the ER is not shuttled to the nucleus as per usual in its functioning state (161). The reduction in cellular ER protein caused by fulvestrant is not due to the downregulation of ER mRNA levels, but rather to the accelerated degradation of the receptor, driven by the instability of the fulvestrant-ER complex. Therefore, fulvestrant exerts its antiestrogen effects through various mechanisms, including the impairment of ER dimerization, the inhibition of ER activity, and the accelerated proteasomal degradation of the ER (21, 158, 160).

## 4 Current progress and future directions

### 4.1 Current progress in tamoxifen treatment

Although there have been numerous clinical trials conducted for tamoxifen use in breast cancer endocrine therapy, a more recent trial known as MONALEESA 7 was conducted with a focus on combination therapy in breast cancer treatment. The MONALEESA 7 trial is a randomized, double-blind, placebo-controlled, international, phase III clinical trial comparing the CDK 4/6 inhibitor, ribociclib, with a placebo or in addition to endocrine therapy such as tamoxifen. Premenopausal and perimenopausal women (n = 672) with HER2-negative, hormone receptor-positive, advanced breast cancer were enrolled in the study between 2014 and 2016 (162). The trial found an estimated overall survival among those who received tamoxifen treatment in the ribociclib group was 71.2% versus 54.5% in the placebo group at 42 months. Furthermore, there was approximately 29% lower risk of death in those receiving ribociclib compared to those receiving endocrine therapy alone (162). Additionally, an update to the study showed that ribociclib plus endocrine therapies, such as tamoxifen, displayed a persistent and significantly longer overall survival compared to endocrine therapy alone when observing the 58.7 vs. 48.0 months timeframes. These results demonstrate a reduction in the relative risk of death by 24% and were consistent with the final analysis for overall survival (163). Therefore, utilizing endocrine therapies such as tamoxifen in combination with other drugs like CDK 4/6 inhibitors are a promising future for the prevention, disease-free maintenance, and treatment of hormone-positive breast carcinomas.

### 4.2 Current progress in toremifene therapy

Several clinical trials for high dose-toremifene treatment have been conducted in Japan and found to be effective as part of “hormone rotation therapy” for the treatment of metastatic breast cancer. Although clinical trials are yet to be conducted regarding the effectiveness of high-dose toremifene therapy against recurrent breast cancer or postmenopausal hormone-sensitive progressive breast cancer, Fushima et al. show promising research in their study of high dose-toremifene for hormone receptor-positive metastatic breast carcinoma with secondary resistance to aromatase inhibitors (112).

### 4.3 Current progress in raloxifene therapy

Various clinical trials, including the MONA trial and the National Surgical Adjuvant Breast and Bowel Project Study (NSABP) of Tamoxifen and Raloxifene (STAR) P-2 trial, have been undertaken regarding the effectiveness and safety of raloxifene in the risk reduction of breast carcinoma in postmenopausal women. However, due to its low bioavailability, the generation of a raloxifene-like drug with an increase in pharmacokinetics was developed (164, 165). This benzothiophene analog of raloxifene is a prodrug known as arzoxifene. However, a phase III trial comparing arzoxifene to tamoxifen as a first-line treatment was terminated when data suggested that arzoxifene was inferior to tamoxifen for the treatment of locally advanced or metastatic breast cancer with respect to a time to progression endpoint (166). Currently, raloxifene is the sole benzothiophene SERM approved by the FDA for use in breast cancer risk reduction. Upon comparing and reviewing data collected from clinical trials, raloxifene is a generally well-tolerated and an effective drug in the risk reduction of breast carcinoma in postmenopausal women with osteoporosis (129, 167).

### 4.4 Current progress in fulvestrant therapy

Recently, the FDA has approved the use of inavolisib, a potent and selective inhibitor of the p110α catalytic subunit of phosphatidylinositol 3-kinase (PIK3CA), plus the CDK4/6 inhibitor palbociclib and the sole FDA-approved SERD, fulvestrant. Inavolisib promotes the degradation of mutated p110α and has been shown to display synergistic activity in combination treatment with palbociclib and fulvestrant for PIK3CA-mutated, hormone receptor (HR) positive, HER2-negative, locally advanced or metastatic breast cancer following relapse on or after the completion of adjuvant endocrine therapy (168, 169). Treatment with inavolisib plus palbociclib-fulvestrant resulted in a longer progression-free survival for patients than those placed on placebo plus palbociclib-fulvestrant, although with greater incidence of toxic effects. Overall, this combination therapy demonstrated good tolerability with a manageable safety profile (168, 169).

### 4.5 Future directions

Alongside the extensive research for the treatment of ER-positive breast cancer with SERMs, SERDs, and AIs, new drug therapies are currently under research and progress in clinical trials. These include the Selective Estrogen Receptor Antagonist/Degrader, Giredestrant, and the Selective Androgen Receptor Modulator (SARM), Enobosarm (170–172). Giredestrant, a non-steroidal, oral, selective ER antagonist and SERD, has shown promise in phase II trials as a single-agent drug therapy to treat locally advanced or metastatic breast cancer with an ER-positive, HER-negative profile (171, 173). In the phase II acelERA BC study, daily 30 mg giredestrant was administered orally to patients until disease progression or unacceptable toxicity for 28-day cycles (171). Following the treatment course, giredestrant displayed a numerical improvement compared to the physician’s choice of endocrine monotherapy. Additionally, in the overall study population, there was an approximate 20% relative reduction in the risk of disease progression or death, which was a favourable benefit for patients with ESR1 m tumours (171). This indicates that giredestrant can target mutant ER-reversing progesterone hypersensitivity more effectively than fulvestrant or AIs, as it is a potent antagonist for targeting this mechanism of endocrine resistance (171, 174). Other novel SERDs that are in varying phases of clinical studies include elacestrant, camizestrant, amcenestrant, imlunestrant, and rintodestrant (173, 175).

Additionally, SARMs like enobosarm show great promise as a novel class of endocrine therapy. Like SERMs, SARMs also exhibit both tissue-dependent agonist and antagonist effects (172). Enobosarm is a first‐in‐class oral SARM that targets the Androgen Receptor (AR), thus inhibiting the growth of AR‐positive, ER‐positive breast carcinoma cells (172). As the AR is expressed in approximately 80%–90% of ER-positive breast carcinomas, SARMs serve as alternative means of endocrine therapy for those patients with AR-positive, ER-positive, metastatic breast cancer (176–182). A phase II clinical trial conducted showed that 9 mg or 18 mg, once daily treatment with enobosarm showed a clinical benefit rate of 32% in the 9‐mg cohort and 29% in the 18‐mg cohort for those enrolled in the study (170, 176). The phase III ARTEST trial is currently underway which will observe enobosarm monotherapy versus an active control of a SERM or exemestane, in patients with metastatic breast cancer of ER‐positive, HER2‐negative, and AR‐positive (≥40% nuclei staining) origin who had progressed on previous therapy with a non-steroidal AI, fulvestrant, and a CDK4/6 inhibitor (172).

Although resistance to hormone therapy develops in 30%–50% of ER-positive breast cancer patients, the utilization of combination therapies provides insight into the treatment of resistant and relapse cases (22). The development of tamoxifen resistance is the result of various underlying mechanisms that are still being explored. These include the activation of the signaling pathways via receptor tyrosine kinases, as well as more recent studies which have found the involvement of cell cycle regulators and transcription factors in tamoxifen treatment resistance (183–188). The activation of the PI3K-PTEN/AKT/mTOR pathway via receptor tyrosine kinases overexpression is believed to be closely related to tamoxifen resistance (185, 186). Additionally, Breast Tumour Kinase (BRK), a non-receptor type tyrosine kinase, has been found to confer resistance to tamoxifen treatment in breast cancer through the regulation of CDK1 tyrosine phosphorylation (187). Further, cell cycle regulators, such as LEM4, have been found to render ER + breast cancer cells resistant to tamoxifen when overexpressed through ERα signaling and activation of the cyclin D-CDK4/6 axis (184). Furthermore, the transcription factor KLF4, has been shown to overcome tamoxifen resistance via suppression of the MAPK signaling pathway (183).

## 5 Conclusion

ER-positive breast cancers are the most common molecular subtype of breast cancer, but this does not imply a dismal prognosis. The use of SERMs and SERDs offers hope and improved survival for those diagnosed with ER-positive breast cancers. However, given the opposing cellular effects of ERα and Erβ, the Erα/ERβ ratio is, therefore, an important determinant of breast cancer behavior and response to endocrine therapy. A higher ERα/ERβ ratio has been linked to increased resistance to SERMs and endocrine therapy, whereas a higher ERβ expression correlates with improved responsiveness to treatment (189). These findings highlight the therapeutic potential of ERβ agonists or strategies that restore ERβ expression to counteract ERα-driven malignancy. Additionally, the recent advancements in expanding the scope of endocrine therapies through the use of SARMs and combination therapies provide further inspiration and optimism for those currently battling ER-positive breast cancers.
